# Association of circulating long non-coding RNA MALAT1 in diagnosis, disease surveillance, and prognosis of acute ischemic stroke

**DOI:** 10.1590/1414-431X20209174

**Published:** 2020-10-21

**Authors:** Hongbo Ren, Feng Wu, Bin Liu, Zhiyuan Song, Dacheng Qu

**Affiliations:** 1 Department of Neurosurgery, HanDan Central Hospital, Handan, China; 2 Department of Neurosurgery, Hebei University of Engineering, Handan, China

**Keywords:** Acute ischemic stroke, lncRNA MALAT1, Inflammation, Survival, Disease severity

## Abstract

We aimed to investigate the association of long non-coding RNA metastasis-associated lung adenocarcinoma transcript 1 (lnc-MALAT1) with acute ischemic stroke (AIS), and its association with disease severity, inflammation, and recurrence-free survival (RFS) in AIS patients. One hundred and twenty AIS patients and 120 controls were recruited. Venous blood samples from AIS patients (within 24 h after symptoms onset) and controls (at entry to study) were collected to detect plasma lnc-MALAT1 expression by real-time quantitative polymerase chain reaction. AIS severity was assessed by the National Institutes of Health Stroke Scale (NIHSS) score. Plasma concentrations of inflammation factors (including C-reactive protein (CRP), tumor necrosis factor α (TNF-α), interleukin (IL)-6, IL-8, IL-10, IL-17, and IL-22) were measured and RFS was calculated. lnc-MALAT1 expression was decreased in AIS patients compared to controls, and it had a close correlation with AIS (AUC=0.791, 95% CI: 0.735-0.846). For disease condition, lnc-MALAT1 expression negatively correlated with NIHSS score and pro-inflammatory factor expression (including CRP, TNF-α, IL-6, IL-8, and IL-22), while it positively correlated with anti-inflammatory factor IL-10 expression. Furthermore, lnc-MALAT1 expression was elevated in AIS patients with diabetes. For prognosis, no statistical correlation of lnc-MALAT1 expression with RFS was found, while a trend for longer RFS was observed in patients with lnc-MALAT1 high expression compared to those with lnc-MALAT1 low expression.

## Introduction

Acute ischemic stroke (AIS), a common disorder caused by embolic or thromboembolic occlusion in an artery supplying the brain, results in irreversible infarction of brain tissue and functional impairment, which causes economic and social burden for families (1–3). Current treatments of AIS focus mainly on recovering cerebral circulation and increasing the likelihood of independent living following AIS. Several restrictions exist in current therapies (such as narrow treatment time window and low recanalization rate), and the prognosis of AIS is still far from satisfactory (4–6). Increasing studies disclose that early identification of AIS risk and prediction of prognosis may help improve treatment outcomes of AIS patients (7,8). Thus, searching for convincing biomarkers that monitor AIS risk and predict prognosis is urgently needed.

Long non-coding RNAs (lncRNAs) are RNA transcripts more than 200 nucleotides and rarely encode proteins, which play essential roles in regulation of protein-coding genes and signaling pathways related to development of diseases (8). lncRNA metastasis-associated lung adenocarcinoma transcript 1 (lnc-MALAT1), located on human chromosome 11q13.1, is abundantly expressed and an evolutionarily conserved lncRNA throughout a variety of mammalian species (7). Accumulating data from basic and clinical studies have disclosed that lnc-MALAT1 could protect ischemia-induced brain microvascular endothelial cells (BMECs), implying that lnc-MALAT1 might be involved in cerebrovascular pathologies in stroke (9,10). Meanwhile, lnc-MALAT1 has been reported to be downregulated and primarily play an anti-inflammatory role in patients with neurological disease as well as with cardiovascular and cerebrovascular disease (11,12). Considering the participation of lnc-MALAT1 in cerebrovascular pathologies of stroke and its anti-inflammation effect on cardiovascular and cerebrovascular diseases, we hypothesized that lnc-MALAT1 might serve as a biomarker for disease risk and progression in AIS via regulating inflammation level, whereas related evidence is seldom reported. Hence, we conducted this study to investigate the correlation of lnc-MALAT1 expression with AIS, and the association of lnc-MALAT1 expression with disease severity, inflammation level, as well as recurrence-free survival (RFS) in AIS patients.

## Material and Methods

### Participants

In total, 120 AIS patients and 120 controls with high stroke risk factors were recruited from our hospital between July 2014 and June 2017. For the AIS patients, screening criteria were as follows: i) primary diagnosis of AIS by clinical presentation, brain non-contrast computed tomography (CT), or diffusion-weighted magnetic resonance imaging, according to the “Guidelines for the Early Management of Patients with Acute Ischemic Stroke: A Guideline for Healthcare Professionals from the American Heart Association/American Stroke Association (AHA/ASA)”(13); ii) more than 18 years of age; iii) admitted to our hospital within 24 h after symptom onset; and iv) able to be regularly followed-up. The exclusion criteria were: i) evidence of intracranial hemorrhage; ii) treated with immunosuppressant within 1 month; iii) suffered from infection (active or in the preceding 14 days of stroke); iv) accompanied by hematological malignancies or solid tumors; and v) women who were pregnant or nursing. As for the controls, all of them had no history of stroke or malignancies and were presented with at least three stroke risk factors, such as hypertension, diabetes mellitus, hyperlipidemia, smoking, etc. The Institutional Review Board of HanDan Central Hospital (China) approved the study protocol, and all participants or their guardians provided written informed consent.

### Data collection and stroke severity assessment

At entry to the study, baseline data of AIS patients were obtained through interviews and medical records, including age, gender, body mass index (BMI), smoking, hypertension, diabetes mellitus, hyperlipidemia, hyperuricemia, and chronic kidney disease (CKD). Hypertension was defined as a history of high blood pressure (≥140/90 mmHg) reported by the respondent or current use of antihypertensive medication; diabetes mellitus was defined as a previous diagnosis, treatment with insulin or oral hypoglycemic medications, fasting plasma glucose ≥126 mg/dL, or glycosylated hemoglobin ≥6.5%; hyperlipidemia was defined as current use of antilipidemic medication, total cholesterol ≥5.70 mmol/L, serum triglycerides ≥1.70 mmol/L, or low-density lipoprotein cholesterol ≥3.10 mmol/L; hyperuricemia was defined as serum uric acid ≥420 μmol/L for men and ≥360 μmol/L for women (14,15). Severity of AIS was assessed within the day of admission by use of the National Institutes of Health Stroke Scale (NIHSS) score (according to the Guidelines for the Early Management of Patients With Acute Ischemic Stroke) (13). The NIHSS score aimed at assessing neurological impairment ranging from 0 to 42, a higher score indicates more serious nerve damage, and the classification of severity was as follows: 0-1 point, normal or near normal; 2-4 points, mild stroke; 5-15 points, moderate stroke; 16-20 points, moderate-severe stroke; 21-42 points, severe stroke. Besides, basic characteristics of controls including age, gender, BMI, smoking, hypertension, diabetes mellitus, hyperlipidemia, hyperuricemia, and CKD were also documented at enrollment.

### Blood sample collection and lnc-MALAT1 determination

Venous blood samples were collected from AIS patients (within 24 h after symptoms onset) and controls using ethylene diamine tetraacetic acid (EDTA) tubes, and were subsequently centrifuged at 1600 *g* for 10 min at 4°C (within 30 min) to acquire supernatant. The supernatant was further centrifuged at 16,000 *g* for 10 min at 4°C to obtain the plasma, which was stored at -80°C for further analysis. lnc-MALAT1 relative expression in the plasma of AIS patients and controls was determined by real-time quantitative polymerase chain reaction (RT-qPCR). AIS patients' CRP concentration in plasma was detected using fully automatic POCT fluorescence immunoassay analyzer (Getein Biotech, China), and the plasma level of inflammatory cytokines including tumor necrosis factor α (TNF-α), interleukin-6 (IL-6), IL-8, IL-10, IL-17, and IL-22 were measured by human enzyme-linked immunosorbent assay (ELISA) kits (Thermo Fisher Scientific, USA) according to the manufacturer's recommendations.

### RT-qPCR

Using QIAamp RNA Blood Mini kit (Qiagen, Germany), total RNA was extracted from plasma samples. Then, reverse transcription to cDNA was performed using PrimeScript™ RT reagent kit (Perfect Real Time) (Takara, China), and qPCR was performed using TB Green™ Fast qPCR Mix (Takara). GAPDH was applied as the internal reference. Primers used in the RT-qPCR were as follows: lnc-MALAT1, forward (5′→3′): TCCTAAGGTCAAGAGAAGTGTCAG, reverse (5′→3′): GTGGCGATGTGGCAGAGAA; GAPDH, forward (5′→3′): TGACCACAGTCCATGCCATCAC, reverse (5′→3′): GCCTGCTTCACCACCTTCTTGA.

### Follow-up

All AIS patients received appropriate treatments as recommended by the 2013 AIS Guidelines (AHA/ASA; 13), and were followed-up regularly or as clinically indicated. The last follow-up date was June 30, 2018, and the median follow-up duration was 25.0 months (range: 1.0-42.0 months). RFS was calculated from the date of hospital admission to the date of recurrence or death.

### Statistical analysis

SPSS 24.0 statistical software (IBM, USA) was used for statistical data processing, and GraphPad Prism 6.01 (GraphPad Software Inc., USA) was applied for graph plotting. Continuous variables are reported as means±SD or median and interquartile range (IQR), and the categorical variables are reported as number (percentage). Differences between groups were determined by Student's *t*-test, Wilcoxon rank-sum test, or chi-squared test. Correlation between variables was analyzed by Spearman rank test. Diagnostic value of variables was assessed by receiver operating characteristic (ROC) curve analysis and the derived area under the curve (AUC) as well as 95% confidence interval (CI). RFS profile is shown by plotting the Kaplan-Meier (K-M) curve, and the difference of RFS between groups was determined by the log-rank test. Factors affecting RFS were analyzed by univariate and multivariate Cox's proportional hazards regression models. All tests were two-sided, and a P value <0.05 was considered statistically significant.

## Results

### Characteristics of AIS patients and controls

No difference of age (P=0.363), gender (P=0.479), and BMI (P=0.159) between AIS patients and controls was observed. Also, the percentage of smoking (P=0.796), hypertension (P=0.421), diabetes mellitus (P=0.182), hyperlipidemia (P=0.296), hyperuricemia (P=0.888), as well as CKD (P=0.360) was similar between the two groups. The detailed information about other baseline characteristics are shown in Table 1.

**Table 1:** Characteristics of acute ischemic stroke (AIS) patients and controls.

Parameters	AIS patients	Controls	P value
Age, years	62.4±12.3	61.1±11.0	0.363
Gender (male/female)	82/38	87/33	0.479
BMI, kg/m^2^	24.6±2.8	24.1±2.8	0.159
Smoking	58 (48.3)	60 (50.0)	0.796
Hypertension	108 (90.0)	104 (86.7)	0.421
Diabetes mellitus	26 (21.7)	18 (15.0)	0.182
Hyperlipidemia	55 (45.8)	47 (39.2)	0.296
Hyperuricemia	35 (29.2)	36 (30.0)	0.888
CKD	20 (16.7)	15 (12.5)	0.360

Data are reported as means±SD or number (percentage) for n=120/group. P<0.05 (Student's *t*-test or chi-squared test). BMI: body mass index; CKD: chronic kidney disease.

### Disease severity and inflammatory factors in AIS patients

Mean NIHSS score and mean values of inflammatory factors are reported in Table 2.

**Table 2:** Disease severity, C-reactive protein (CRP), and inflammatory cytokines of acute ischemic stroke patients.

Parameters	Mean	SD	Median	IQR
NIHSS score	7.8	3.5	7.0	5.0–10.0
CRP, mg/L	35.3	21.6	29.0	23.2–38.7
TNF-α, pg/mL	79.5	56.1	60.1	44.9–94.6
IL-6, pg/mL	51.7	29.9	44.7	38.1–54.3
IL-8, pg/mL	62.0	35.1	50.4	40.2–76.4
IL-10, pg/mL	23.3	12.6	20.9	14.1–30.9
IL-17, pg/mL	101.9	49.1	96.2	65.3–122.7
IL-22, pg/mL	85.8	64.9	67.8	48.5–96.9

SD: standard deviation; IQR: inter-quartile range; NIHSS: National Institutes of Health Stroke Scale; TNF-α: tumor necrosis factor α; IL: interleukin.

### Comparison of lnc-MALAT1 expression between AIS patients and controls

**Figure 1:**
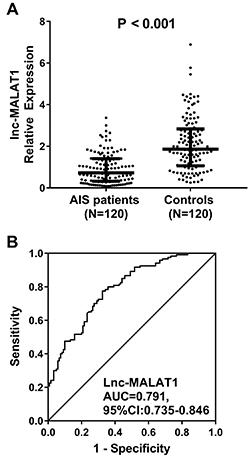
lnc-MALAT1 relative expression in acute ischemic stroke (AIS) patients and controls. Horizontal lines indicate means±SD (Wilcoxon rank sum test) (**A**). Diagnostic value of lnc-MALAT1 for AIS was assessed by ROC curve (**B**). lnc-MALAT1: long non-coding RNA metastasis-associated lung adenocarcinoma transcript 1; ROC curve: receiver operating characteristic curve; AUC: area under the curve.

lnc-MALAT1 expression was decreased in AIS patients compared to controls (P<0.001) (Figure 1A). The ROC curve showed that lnc-MALAT1 had a close correlation with AIS (AUC=0.791, 95%CI: 0.735-0.846) (Figure 1B).

### Correlation of lnc-MALAT1 expression with NIHSS score in AIS patients

**Figure 2:**
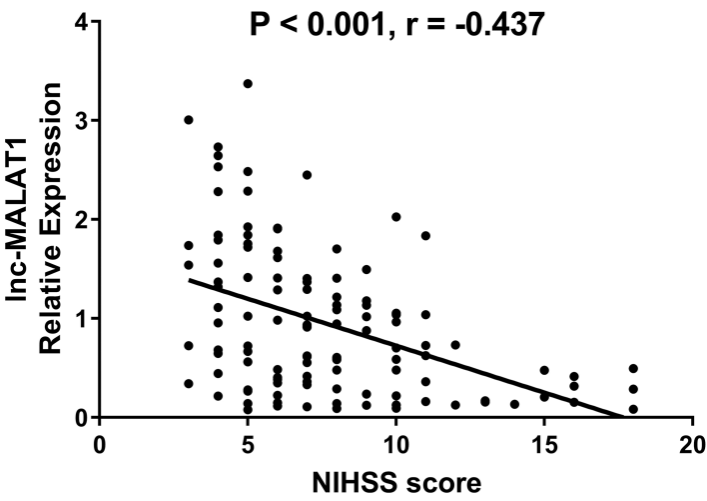
lnc-MALAT1 high expression was correlated with decreased NIHSS score (Spearman rank test). lnc-MALAT1: long non-coding RNA metastasis-associated lung adenocarcinoma transcript 1; NIHSS score: National Institutes of Health Stroke Scale score.

lnc-MALAT1 expression was negatively correlated with NIHSS score in AIS patients (P<0.001, r=-0.437) (Figure 2).

### Correlation of lnc-MALAT1 expression with inflammatory factors in AIS patients

**Figure 3:**
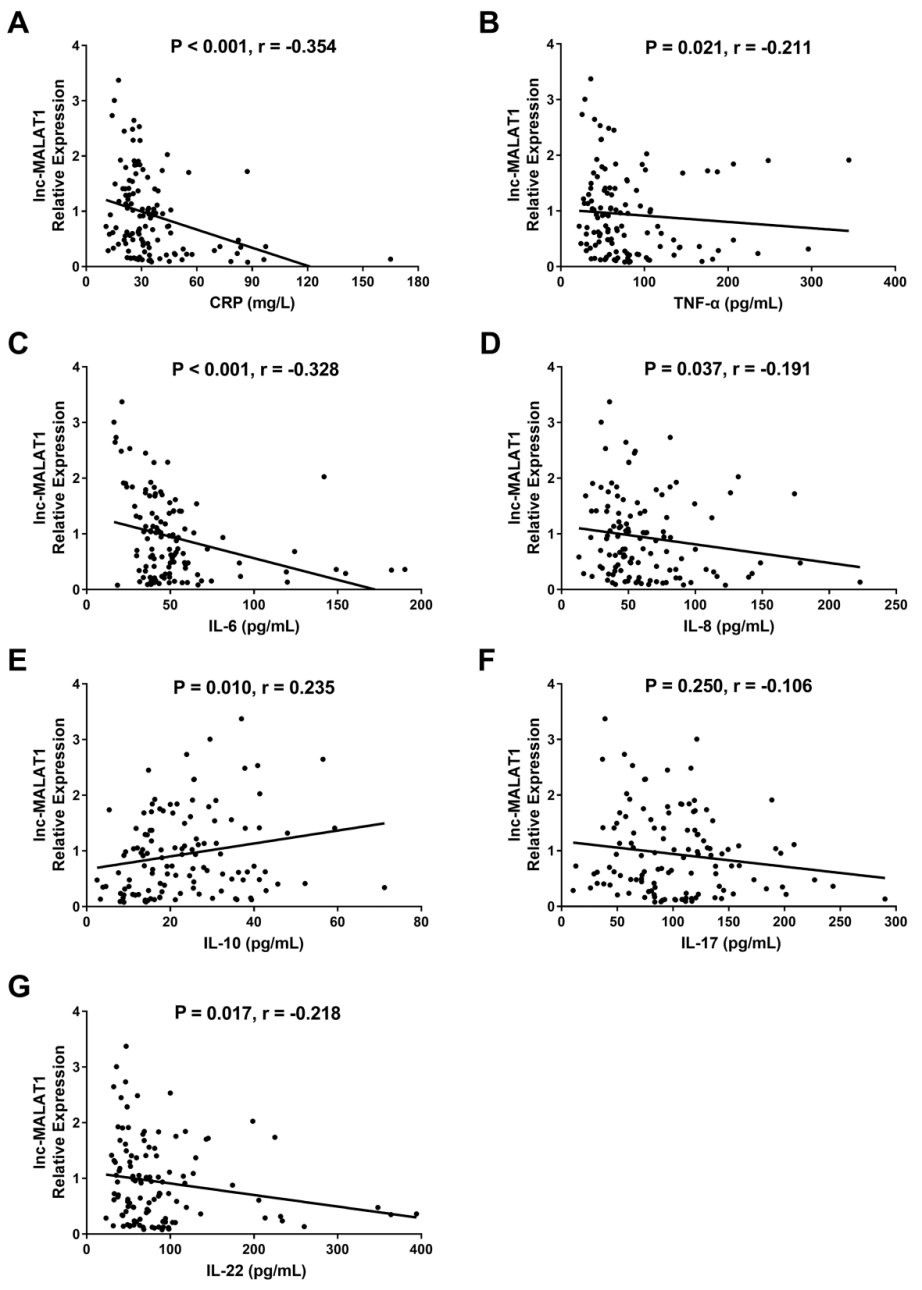
Correlation of lnc-MALAT1 expression with C-reactive protein (CRP) (**A**), tumor necrosis factor α (TNF-α) (**B**), interleukin (IL)-6 (**C**), IL-8 (**D**), IL-10 (**E**), IL-17 (**F**), and IL-22 (**G**) was determined by the Spearman rank test. lnc-MALAT1: long non-coding RNA metastasis-associated lung adenocarcinoma transcript 1.

Lnc-MALAT1 expression was negatively associated with expression of inflammatory factors including CRP (P<0.001, r=-0.354) (Figure 3A), TNF-α (P=0.021, r=-0.211) (Figure 3B), IL-6 (P<0.001, r=0.328) (Figure 3C), IL-8 (P=0.037, r=-0.191) (Figure 3D), and IL-22 (P=0.017, r=-0.218) (Figure 3G), while it was positively correlated with expression of anti-inflammatory cytokine IL-10 (P=0.010, r=0.235) (Figure 3E) in AIS patients. Furthermore, no correlation of lnc-MALAT1 expression with IL-17 expression (P=0.250, r=-0.106) (Figure 3F) was found.

### Correlation of lnc-MALAT1 expression with some risk factors for AIS

**Figure 4:**
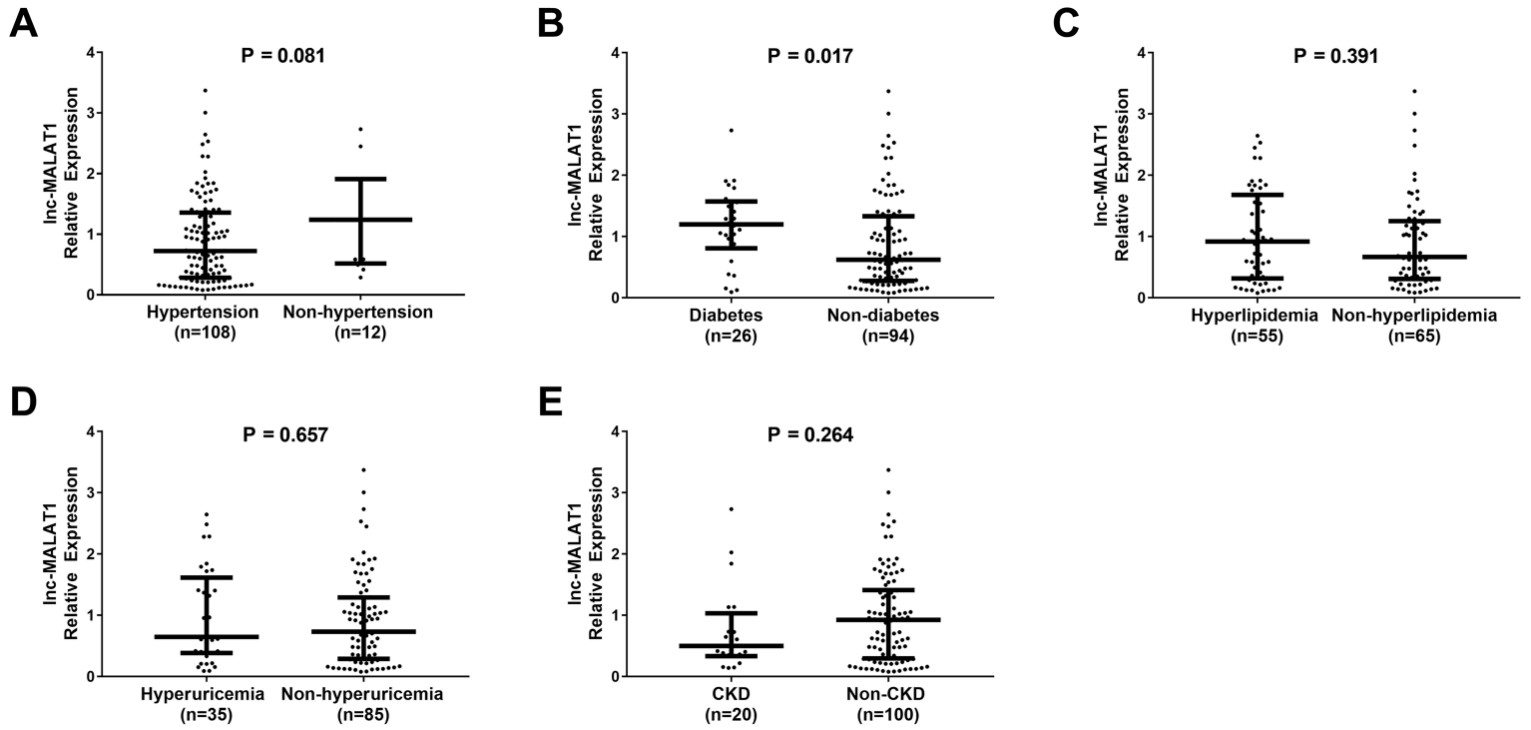
lnc-MALAT1 expression in acute ischemic stroke patients with different risk factors: hypertension and non-hypertension patients (**A**), diabetes and non-diabetes patients (**B**), hyperlipidemia and non-hyperlipidemia patients (**C**), hyperuricemia and non-hyperuricemia patients (**D**), as well as chronic kidney disease (CKD) and non-CKD patients (**E**). Horizontal lines indicate means±SD (Wilcoxon rank sum test). lnc-MALAT1: long non-coding RNA metastasis-associated lung adenocarcinoma transcript 1.

To investigate the correlation of lnc-MALAT1 expression with some risk factors for AIS, all patients were categorized into different subgroups according to the risk factors (Figure 4). Lnc-MALAT1 expression was elevated in diabetes compared to non-diabetes (P=0.017) (Figure 4B). Notably, although no difference of lnc-MALAT1 expression with hypertension was observed, there was a trend for reduced hypertension in lnc-MALAT1 high expression patients compared to lnc-MALAT1 low expression (P=0.081) (Figure 4A). No difference of lnc-MALAT1 expression was found between hyperlipidemia patients and non-hyperlipidemia patients (P=0.391) (Figure 4C), hyperuricemia patients and non-hyperuricemia patients (P=0.657) (Figure 4D), or CKD patients and non-CKD patients (P=0.264) (Figure 4E). These data indicated that lnc-MALAT1 expression was positively correlated with diabetes occurrence in AIS patients. Additionally, no correlation of lnc-MALAT1 expression with risk factors including hypertension (P=0.757), diabetes mellitus (P=0.082), hyperlipidemia (P=0.232), hyperuricemia (P=0.740), and CKD (P=0.642) was found in the control group (Supplementary Table S1). However, a trend for increased lnc-MALAT1 expression was observed in diabetes mellitus patients compared to non-diabetes mellitus patients in the control group.

### Comparison of RFS between lnc-MALAT1 high expression patients and lnc-MALAT1 low expression patients

**Figure 5:**
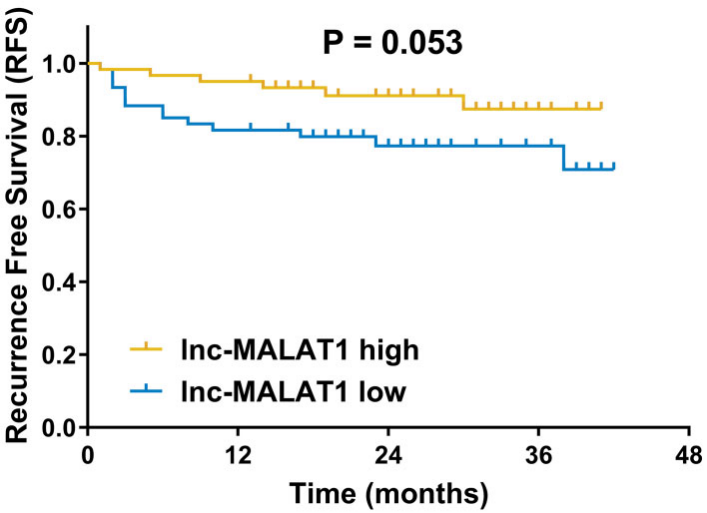
Kaplan-Meier curves show the recurrence-free survival in lnc-MALAT1 high expression and lnc-MALAT1 low expression patients determined by the log-rank test. lnc-MALAT1: long non-coding RNA metastasis-associated lung adenocarcinoma transcript 1.

Patients were divided into lnc-MALAT1 high expression group and lnc-MALAT1 low expression group according to the median value of lnc-MALAT1 expression. No difference of RFS was found between the groups, while there was a trend for longer RFS in lnc-MALAT1 high expression (P=0.053) compared to lnc-MALAT1 low expression (Figure 5).

### Analysis of factors affecting RFS in AIS patients

Univariate Cox's regression analysis showed that there was a trend for better RFS in lnc-MALAT1 high expression patients compared to lnc-MALAT1 low expression patients (although statistically non-significant) (P=0.063), while CRP (≥29.0 *vs* <29.0 mg/L) was associated with worse RFS (P=0.006) (Table 3). Moreover, multivariate Cox's regression analysis showed that lnc-MALAT1 high expression was not an independent predictive factor for RFS, whereas CRP (≥29.0 *vs* <29.0 mg/L) independently predicted reduced RFS (P=0.024).

**Table 3:** Factors affecting recurrence-free survival by Cox's proportional hazards regression model analyses.

Items	Univariate Cox's regression		Multivariate Cox's regression	
	P value	HR (95%CI)	P value	HR (95%CI)
lnc-MALAT1 (high *vs* low)	0.062	0.402 (0.154–1.047)	0.200	0.482 (0.157–1.473)
Age (≥61 *vs* <61 years)	0.976	0.986 (0.406–2.397)	0.974	0.981 (0.307–3.137)
Gender (male/female)	0.853	1.095 (0.421–2.850)	0.965	0.974 (0.304–3.120)
BMI (≥24.2 *vs* <24.2 kg/m^2^)	0.506	0.741 (0.307–1.791)	0.725	0.835 (0.305–2.282)
Smoking (yes *vs* no)	0.456	0.712 (0.291–1.741)	0.509	0.726 (0.281–1.876)
Hypertension (yes *vs* no)	0.836	1.167 (0.270–5.047)	0.704	1.371 (0.269–6.980)
Diabetes mellitus (yes *vs* no)	0.193	0.378 (0.088–1.633)	0.526	0.585 (0.111–3.075)
Hyperlipidemia (yes *vs* no)	0.853	0.920 (0.380–2.227)	0.978	1.015 (0.365–2.821)
Hyperuricemia (yes *vs* no)	0.958	1.026 (0.394–2.673)	0.712	0.819 (0.283–2.370)
CKD (yes *vs* no)	0.821	1.136 (0.377–3.418)	0.609	1.435 (0.360–5.728)
NIHSS score (≥7 *vs* <7)	0.428	0.701 (0.292–1.687)	0.229	0.557 (0.215–1.445)
CRP (≥29.0 *vs* <29.0 mg/L)	**0.006**	4.683 (1.563–14.028)	**0.024**	4.720 (1.224–18.197)
TNF-α (≥60.1 *vs* <60.1 pg/mL)	0.126	2.052 (0.818–5.151)	0.350	1.879 (0.501–7.041)
IL-6 (≥44.7 *vs* <44.7 pg/mL)	0.301	1.605 (0.655–3.931)	0.899	1.069 (0.381–2.996)
IL-8 (≥50.4 *vs* <50.4 pg/mL)	0.988	0.993 (0.413–2.387)	0.387	0.559 (0.149–2.088)
IL-10 (≥20.9 *vs* <20.9 pg/mL)	0.133	0.493 (0.196–1.239)	0.548	0.707 (0.227–2.195)
IL-17 (≥96.2 *vs* 96.2 pg/mL)	0.586	0.783 (0.324–1.889)	0.153	0.474 (0.170–1.319)
IL-22 (≥67.8 *vs* <67.8 pg/mL)	0.299	1.608 (0.656–3.942)	0.716	0.797 (0.235–2.705)

HR: hazard ratio; CI: confidence interval; BMI: body mass index; CKD: chronic kidney disease; NIHSS: National Institutes of Health Stroke Scale; CRP: C-reactive protein; TNF-α: tumor necrosis factor α; IL: interleukin. Significant P values are shown in bold.

## Discussion

Our results indicated that: 1) lnc-MALAT1 expression was decreased in AIS patients compared to controls, and it had a close correlation with AIS; 2) lnc-MALAT1 high expression was correlated with decreased NIHSS score as well as reduced inflammatory factors levels in AIS patients; and 3) lnc-MALAT1 high expression showed a trend for prolonged RFS compared to lnc-MALAT1 low expression in AIS patients, but without statistical significance.

lncRNAs are involved in a variety of biological processes (such as gene transcription, organizing RNA-protein complex, and management in protein activity) (16–19). As one of the frequently investigated lncRNAs, lnc-MALAT1 has various molecular functions (including alternative splicing, transcriptional regulation, and post-transcriptional regulation), and also participates in multiple physiological functions (including neural development, skeletal myogenesis, and vascular growth) (7). As to its role in neurological diseases or cerebrovascular diseases, previous studies demonstrate that lnc-MALAT1 presents protective effects in these diseases via repressing pro-apoptotic or pro-inflammatory factors (10,11,20,21). For example, one study reported that lnc-MALAT1 protects human brain vascular endothelial cells from OGD-induced apoptosis through activating phosphatidylinositol 3-kinase (PI3K) (21). In addition, lnc-MALAT1 expression is decreased in the spinal cords of mice with experimental autoimmune encephalomyelitis, and its knockdown raises the level of inflammatory cytokines (including IL-1 and IL-6) (11). Also, silencing of lnc-MALAT1 raises the level of pro-apoptotic factor Bim and pro-inflammatory cytokines (including MCP-1 as well as E-selectin) in BMECs following oxygen-glucose deprivation (OGD), which is an *in vitro* mimic of ischemic stroke conditions (10). Another study discloses that lnc-MALAT1 knockdown aggravates OGD-induced overexpression of pro-inflammatory cytokines including MCP-1 and IL-6 in mouse cerebral microvascular endothelial cells (20). These studies reveal that lnc-MALAT1 has a protective effect in neurological and cerebrovascular diseases through reducing inflammation or inhibiting cell apoptosis.

Previous clinical trials focus mainly on the exploration of lnc-MALAT1 in cancer patients, showing that lnc-MALAT1 plays a tumor-promotive role, while information about the role of lnc-MALAT1 in neurological diseases or cerebrovascular diseases is still limited (22–24). Only a few studies show that lnc-MALAT1 expression is reduced in central nervous system tissues from multiple sclerosis patients and carotid plaques from atherosclerosis patients (11,12). As for the correlation of lnc-MALAT1 with disease severity and inflammation in neurological and cerebrovascular disease patients, only one study shows that elevated lnc-MALAT1 expression is correlated with less advanced lesions in atherosclerosis patients (12). Considering that lnc-MALAT1 might participate in the cerebrovascular pathologies of stroke according to previous studies, and it presented an anti-inflammatory effect in cardiovascular and cerebrovascular diseases, we hypothesized that lnc-MALAT1 expression might be related to disease risk, progression, or inflammation level of AIS (9–12,25).

In our study, we assessed the association of lnc-MALAT1 with AIS, and observed that lnc-MALAT1 expression was lower in AIS patients compared to controls, and lnc-MALAT1 expression presented good diagnostic value for AIS, which might be due to its protective effect via repressing cell apoptosis of BMECs in these diseases. Moreover, we investigated the correlation of lnc-MALAT1 expression with disease severity as well as inflammation in AIS patients. We observed that lnc-MALAT1 high expression was associated with decreased NIHSS score and reduced level of inflammatory factors (including CRP, TNF-α, IL-6, IL-8, and IL-22). In addition, there was a trend for an increased proportion of diabetes (without statistical significance) in AIS patients with lnc-MALAT1 high expression. The possible reasons for these results might be as follows: 1) lnc-MALAT1 had a protective effect on brain microvascular endothelial cells through inhibiting cell apoptosis, thereby contributed to maintaining healthy brain endothelium that was essential for normal cerebrovascular physiology, therefore the severity of AIS was attenuated and a decreased NIHSS score was observed (7,20); 2) lnc-MALAT1 might decrease the expression of transcriptional factor nuclear factor-kappa B (NF-κB) that drove the transcription of a series of inflammatory factors, thus led to reduced inflammatory factor level and alleviated inflammation in AIS patients (26); 3) lnc-MALAT1 protected the brain microvascular endothelial cells against apoptosis, thereby facilitated microvascular permeability and helped restore cerebral vasoreactivity, thus it might attenuate hypertension in AIS patients (25); and 4) lnc-MALAT1 was found to interact with transcription factor Foxo1 and SIRT1 transcription to induce poor glycemic control and insulin resistance, which might promote the occurrence of diabetes (27,28).

As to the prognostic value of lnc-MALAT1 in human diseases, it has been identified as a valuable biomarker for disease prognosis in several cancers, while limited clinical studies have been found in AIS. Only one study reports that lnc-MALAT1 high expression is associated with prolonged main adverse cardiovascular and cerebrovascular events (MACCE)-free survival in atherosclerosis patients (12). To our knowledge, there is still no evidence about the influence of lnc-MALAT1 on the prognosis in AIS patients. In our study, we observed that there was a trend for better RFS in lnc-MALAT1 high expression AIS patients, but without statistical significance. These results might be due to: 1) lnc-MALAT1 reduced the production of inflammation cytokines, further decreased inflammation, and attenuated disease progression, thus it led to better RFS in AIS patients (10,20); 2) lnc-MALAT1 might facilitate sensitivity to treatment in AIS, thereby it increased treatment efficacy and resulted in longer RFS in AIS patients.

Some limitations existed in our study: 1) the sample size (120 AIS patients) was relatively small, thus the statistical power might be low; 2) the median follow-up duration (25.0 months (range: 1.0-42.0 months)) was relatively short, thus the correlation of lnc-MALAT1 expression with long-term prognosis remains to be further investigated; and 3) detailed mechanism of lnc-MALAT1 in AIS is still unclear, and further study is still needed.

In conclusion, lnc-MALAT1 was downregulated and presented a close association with AIS, and its high expression correlated with decreased NIHSS score as well as reduced inflammation in AIS patients. Moreover, there was a trend for better RFS in AIS patients with lnc-MALAT1 high expression compared to those with lnc-MALAT1 low expression, despite the lack of statistical correlation.

## Supplementary Material

Click here to view [pdf].
